# Antimicrobial and Adjuvant Potencies of Di-*n*-alkyl Substituted Diazalariat Ethers

**DOI:** 10.3390/antibiotics12101513

**Published:** 2023-10-05

**Authors:** Mohit B. Patel, Helena Spikes, Robert S. Bailey, Thomas Connell, Hannah Gill, Michael R. Gokel, Rebecca Harris, Joseph W. Meisel, Saeedeh Negin, Shanheng Andrew Yin, George W. Gokel

**Affiliations:** Department of Chemistry and Biochemistry, University of Missouri-St. Louis, St. Louis, MO 63121, USA

**Keywords:** adjuvant, antibiotic, antibiotic resistance, bacteria, crown ether, cytotoxicity, efflux pumps, lariat ether, membrane permeability, resistance reversal

## Abstract

Lariat ethers are macrocyclic polyethers—crown ethers—to which sidearms are appended. 4,13-Diaza-18-crown-6 having twin alkyl chains at the nitrogens show biological activity. They exhibit antibiotic activity, but when co-administered at with an FDA-approved antibiotic, the latter’s potency is often strongly enhanced. Potency enhancements and resistance reversals have been documented in vitro for a range of Gram-negative and Gram-positive bacteria with a variety of antimicrobials. Strains of *E. coli* and *Staphylococcus aureus* having resistance to a range of drugs have been studied and the potency enhancements (checkerboards) are reported here. Drugs included in the present study are ampicillin, cefepime, chlortetracycline, ciprofloxacin, doxycycline, kanamycin, minocycline, norfloxacin, oxycycline, penicillin G, and tetracycline. Enhancements of norfloxacin potency against *S. aureus* 1199B of up to 128-fold were observed. The properties of these lariat ethers have been studied to determine solubility, their membrane penetration, cytotoxicity and mammalian cell survival, and their effect on bacterial efflux pumps. It is shown that in some cases, the lariat ethers have complex antimicrobials with considerable selectivity. Based on these observations, including 1:1 complexation between lariat ethers and antimicrobials and the cytotoxicity of the MeI salts showing a separation index of 32-fold, they hold significant potential for further development.

## 1. Introduction

Although the COVID-19 viral pandemic has dominated the news during the past several years, the importance of the antibiotic resistance crisis to worldwide human health remains critical and requires little introduction [1,2,3]. Both the Centers for Disease Control and Prevention (CDC) [4] and the World Health Organization (WHO) have issued various reports expressing urgent concern. The problem is both increasing resistance and the dearth of novel antimicrobials in current drug pipelines [5,6]. The search in nature for new structures having antimicrobial activity remains an important approach to finding new antibiotics. Another approach involves the synthesis of new derivatives of known drugs or even of novel chemical structures—often compounds inspired by antimicrobial peptides [7,8]. The goals are to enhance potency, to minimize mammalian cytotoxicity, to overcome resistance, to minimize drug interactions, or all four. Recent work in developing macrocycles [9], peptides [10,11], peptoids [12], and cyclic peptides [13] exemplify the “novel structure” approach. Various crown ethers have also shown a range of biological activity [14,15,16,17,18,19,20,21,22,23] against microbes and mammals, as have a variety of natural alternatives [24].

Amphiphilic peptides [25,26,27,28,29,30] comprise an important class of antimicrobials. These are often isolated from bacteria, frogs, and a range of other species [31]. In recent years, a broad family of synthetic compounds known as peptoids [32] has emerged and many of these have both amphiphilic and antimicrobial properties [33,34,35,36,37]. The amphiphiles that disrupt or enhance the permeability of membranes [38,39,40] are often effective antimicrobials and typically exhibit a low rate of resistance development. As such, amphiphiles present an attractive target for antimicrobial drug development. Colistin (polymyxin E) [41] and daptomycin [42] were both isolated from bacteria and are good examples of natural amphiphilic antimicrobials.

Hydraphiles are amphiphiles that possess linked crown ether rings and are terminated by a variety of sidearms. They may be represented using the shorthand H<N18N>H for diaza-18-crown-6 as R^1^<N18N>R<N18N>R<N18N>R^1^. In this family, R is typically dodecylene or tetradecylene and R^1^ is benzyl. Many hydraphiles function as unimolecular ion channels [43]. These synthetic ionophores function as antibiotic adjuvants against multidrug resistant bacteria [44]. This is accomplished by penetration of the amphiphile into the bacterium, partially disrupting membrane permeability and inhibiting the efflux pump function [45]. The hydraphiles perform these functions effectively at no more than half their minimum inhibitory concentrations (MIC) [46].

A simpler family of biologically active dialkylmacrocycles is known as lariat ethers (LEs). These diazacrown ether derivatives are non-peptidic synthetic amphiphiles. Lariat ethers were originally developed to have donor-rich sidearms that could help to envelop a cation bound within the macrocycle. This broad family of three-dimensional binders has been studied extensively for their ability to bind and transport cations [47]. The compounds that are the subject of this report are lariat ethers having alkyl sidearms that lack donor groups. Generally, they are *N,N*′-di-*n*-alkyl-4,13-diaza-18-crown-6 compounds, which may be represented schematically as R<N18N>R or C_n_<N18N>C_n_ where “n” is the number of carbons in a linear alkyl sidearm. Some of these dialkyldiaza lariat ethers have been assayed and shown to possess antimicrobial properties [48].

The lariat ethers that contain sidearm donors function as carriers. The dialkyl lariat ethers do so as well [49], but several compounds in the dialkyl lariat ether family form cation conducting channels [45] and have significant biological activity. When co-administered with FDA-approved antimicrobial drugs, C_8_LE [C_8_H_17_<N18N>C_8_H_17_] and C_11_LE [C_11_H_23_<N18N>C_11_H_23_] sidearmed lariat ethers increased the potencies of antibiotics against drug-sensitive *Escherichia coli*. For example, rifampicin was 20-fold more potent against *E. coli* when administered with ½ MIC of C_8_LE (**7**).

Based on the above observations and previously obtained results, we hypothesized that the lariat ethers—dialkyl sidearmed macrocycles—form aggregates within the boundary membranes of bacteria. If this hypothesis is correct, membrane permeability will be enhanced and ion homeostasis should be affected. Increased permeability could enhance drug penetration and disruption of ion homeostasis would encumber any ion-regulated cellular defense response.

It was found previously that within the R<N18N>R family, where R was *n*-alkyl having an even number of carbons, that C_10_LE (C_10_<N18N>C_10_) was the most efficient sodium transporter through liposomal membranes, followed by C_12_ and C_8_LEs [48]. Further, only C_10_ (**1**–**3**) and C_12_LEs (**4**–**6**) showed significant activity against Gram-positive *Bacillus subtilis*. Of these, just C_10_LE (**1**) was active against Gram-negative K12 *E. coli*. The LE having *n*-undecyl side chains also proved active, albeit somewhat less so than C_10_LE. We therefore explored the ability of C_10_ and C_12_ lariat ethers to function as adjuvants to recover the potency of norfloxacin against resistant *Staphylococcus aureus* 1199B bacteria. *S. aureus* 1199B is a Gram-positive bacterium that overexpresses the NorA efflux pump [50]. This protein exports norfloxacin by a mechanism involving a Na^+^ antiport. Hence, the disruption of either the membrane or the regulated cation gradient, or both, should diminish a resistance response and enhance antimicrobial potency.

In an effort to increase both the stability and solubility of C_10_ and C_12_ LEs, we prepared the corresponding hydrochloride and methyl iodide salts. The salts were tested for enhanced solubility compared to the free bases. The combination of higher solubility and positive charges was expected to increase antimicrobial potency. Positively charged amphiphiles should have a higher affinity for the negative lipopolysaccharide bacterial surface than do the corresponding neutral compounds.

Both hydrochloride and methiodide salts of LEs would affect the charge state of the macro ring. A difference between them is that protonation is reversible. In vitro, protonation and re-protonation is likely because the pKa values for the macro ring nitrogen atoms in di-*n*-butyl-diaza-18-crown-6 are pK_1_ = 9.40, pK_2_ 7.97. Protonation and re-protonation of the ring nitrogen atoms would permit the sidearm orientations to vary. In principle, this would permit the sidearms to adapt conformationally to different steric constraints. In contrast, the positive charges in the methiodide salts are permanent. In either case, the positive charges will diminish the cation complexing ability of the macrocycle. In the cases of the methiodide salts, the two methyl groups could also add a steric impediment to cation binding. We describe herein the results of studies that attempt to determine the import of protonation and methylation on bacterial growth inhibition and/or antimicrobial potency recovery.

## 2. Results and Discussion

### 2.1. The Compounds and Organisms

**Figure 1:**
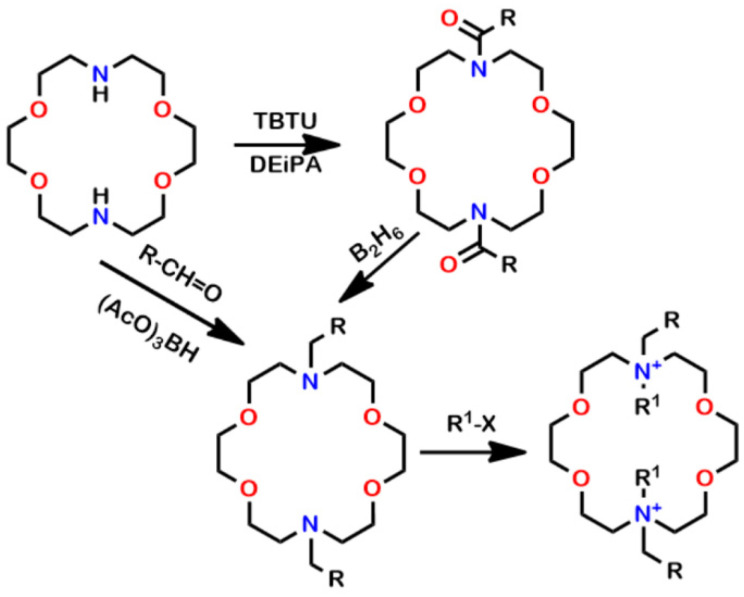
Syntheses of the lariat ethers and salts. The eventual sidearms (CH_2_R) are *n*-octyl, *n*-decyl (**1**, **2**, **3**), *n*-undecyl, *n*-dodecyl (**4**, **5**, **6**), and *n*-tetradecyl. R^1^ is either H or CH_3_. (**1**, **4**) The salts (R^1^) are hydrochlorides (**2**, **5**) or methiodides (**3**, **6**). See the experimental section for additional details.

Figure 1 shows the chemical structures and preparation of the compounds that were used in the study. 4,13-Diaza-18-crown-6 was alkylated and reduced or the sidearms were attached by reductive amination. The bacterium we call *E. coli* tet^R^ was prepared in our laboratory and has a MIC against tetracycline of 1000 µM [51].

Derivatization of 4,13-diaza-18-crown-6 was accomplished either by forming the *bis*(amide) and reducing it or by reductive amination, both illustrated in Figure 1. The hydrochloride salts were formed by treatment with HCl in dioxane. Reaction of the LEs with CH_3_I in diethyl ether or toluene afforded the *bis*(methiodide) salts. The compounds illustrated in Figure 1 are C_10_LE (**1**, n = 10), C_10_LE•2HCl (**2**), C_10_LE•2MeI (**3**), C_12_LE (**4**, n = 12), C_12_LE•2HCl (**5**), and C_12_LE•2MeI (**6**).

### 2.2. Lariat Ethers and Derivatives’ Activities against E. coli DH5α

The MIC data shown in Table 1 are for C_8_ to C_14_LE derivatives against the non-pathogenic DH5α strain of *E. coli* in the absence of any additional antimicrobial agent. The MICs shown in the table are artificially limited to 128 µM. Higher concentrations than this can be measured, but at such low potencies for bacteriostatic action, we deem the amphiphile to be inactive. Thus, C_8_LE (**7**) was only marginally active against K12 *E. coli* (MIC = 100 µM) and it was even less active (MIC = 206 µM) against the DH5α strain (data not in Table 1). In general, the MIC results were in concert with earlier reports. Table 1 shows previously reported data for the potencies of lariat ethers having *n*-octyl, *n*-undecyl, and *n*-tetradecyl side chains [48]. Additional data are for lariat ether hydrochlorides and lariat ether methiodides against DH5α *E. coli*. Since we considered MICs higher than 128 µM as inactive, any MIC higher than that value is reported as 128 µM irrespective of the actual value. C_11_LE free base is included for comparison in the table, but the protonated and methylated forms were not studied.

An earlier potency study [48] of an *N,N*′-di-*n*-alkyl-4,13-diaza-18-crown-6 family of analogs having sidearms of 8, 10, 12, 14, 16, and 18 carbons was conducted against *E. coli, Bacillus subtilis*, and the fungus *Saccharomyces cerevisiae*. In this study, activity against DH5α *E. coli* was observed only with C_10_LE (**1**). C_10_LE (**1**) and C_12_LE (**4**) were active against Gram-positive *B. subtilis* and *S. cerevisiae*, but C_12_LE (**4**) was not active against *E. coli.* The *E. coli* data shown in Table 1 are consistent with these published results. Remarkably, C_12_LE•2MeI (**6**) is active against *E. coli*, but the dihydrochloride C_12_LE•2HCl (**5**) is not. The surprising contrast in biological responses of these closely related compounds engendered a study of the physical chemical variables that might account for the differences.

### 2.3. Solubility of Lariat Ethers and Salts

Water solubility is required for a drug to circulate in the bloodstream. Acidic or basic drugs are often converted into salts in the administering formulation to enhance solubility [52]. The present results relate to bacterial cells rather than infection in a complex organism. The solubilities of lariat ethers **1**–**6** were determined in phosphate-buffered saline (PBS) and Mueller Hinton II broth [53] (MHII includes protein, starch, Mg^2+^, and Ca^2+^, at pH 7.3, purchased from Millipore Sigma, Burlington, MA, USA) media [54] at concentrations from 1 µM to 128 µM. The compounds were dissolved in DMSO at 25.6 mM and serially diluted by 2-fold to a minimum concentration of 200 nM. The compound was added to a microdilution containing either MHII media or PBS such that the final concentration of DMSO was ≤0.5% [55]. The absorbance of the whole plate was determined at 540 nm after 2 h of incubation at 37 °C. Any absorbance greater than that of the average blank plus three times its standard deviation was considered to be turbidity resulting from insoluble matter. The average solubility results from three replicates in each case are shown in Table 2. Verapamil hydrochloride and reserpine were used as high- and low-solubility controls, respectively.

**Figure 2:**
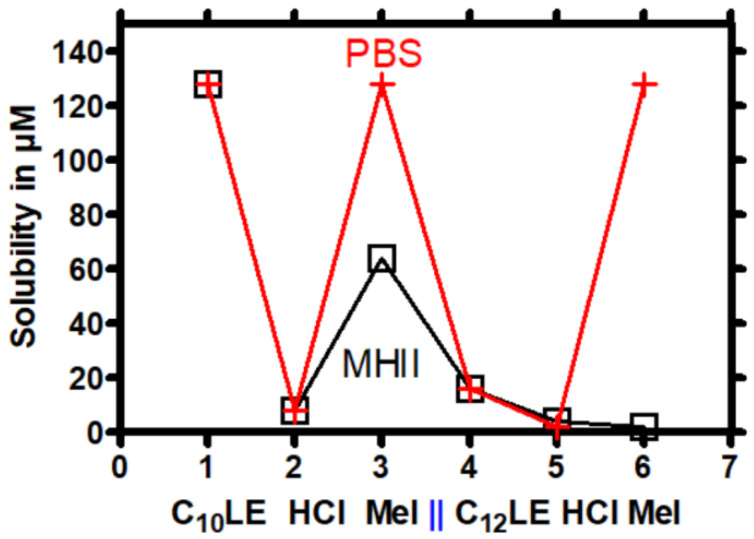
Solubilities of C_10_LE (**1**), C_10_LE•2HCl (**2**), C_10_LE•2MeI (**3**), C_12_LE (**4**), C_12_LE•2HCl (**5**), and C_12_LE•2MeI (**6**) in PBS or MHII media. Open squares correspond to MHII media and crosses refer to PBS media. The limit of solubility measurements was arbitrarily set at 128 μM.

As expected from published data [56], verapamil hydrochloride was soluble in water as well as in media, while reserpine was soluble only at 16 µM in both PBS and MHII. We surveyed both the HCl and MeI salts of C_10_LE (**1**) and C_12_LE (**4**). As shown in Table 2, the methyl iodide (MeI) salts of C_10_ and C_12_ lariat ethers showed greater solubility in PBS than do the corresponding HCl salts. The free base form of C_10_LE (**1**), was more soluble in both PBS and MHII than was C_12_LE (**4**). This was expected, since C_10_LE (**1**) contains four fewer carbons than C_12_LE (**4**). It is interesting to note that the solubilities of methiodide salts **3** and **6** were greater in PBS than in MHII. Although MHII is a more complex medium than phosphate-buffered saline, the solubilities showed a similar profile except for C_12_LE•2MeI (**6**). Figure 2 shows the data of Table 2 in graphical form.

The difference in solubilities between C_10_LE•2MeI (**3**) and C_12_LE•2MeI (**6**) in MH II media was surprising, but reproducible. We speculate that the explanation lies in a difference in sidearm orientations. The solid state structure for a sodium complex of C_12_LE (**4**) has been reported [57]. In it, the side chains are parallel and in contact in the crystal. To our knowledge, no structure is available for a lariat ether hydrochloride or methiodide salt. We have been unsuccessful in obtaining crystals of either. It seems reasonable to think that the sidearms in the bis(methiodide) are on opposite sides of the macrocycle, which would relieve strain engendered by the methyl groups attached to the macro ring. If the sidearms were aligned in C_10_LE (**1**) and splayed in C_12_LE (**4**), the former’s more concentrated hydrophobic surface could reduce its solubility compared to splayed sidearms. Of course, this difference is manifested only in MHII and not in PBS media.

The macro ring portion of lariat ethers can bind various cations, albeit with modest affinity in aqueous solution. In the presence of Na^+^, K^+^, or Ca^2+^, the LE free base compounds form salt complexes that are polar and more soluble than the neutral precursors. As a result, their solubilities as complexes are ≥128 µM, the limit of the measurements made. The binding of either C_10_LE (**1**) or C_12_LE (**4**) salts will be diminished whether hydrochlorides or methiodides owing to the positive charges in the macro ring. The charge state of the hydrochloride salt will depend on pH, but the positive charges in **2**, **3**, **5**, and **6** should increase their affinities for the negative bacterial surface. The lariat ethers may also exhibit affinity towards proteins (non-specific binding).

**Figure 3:**
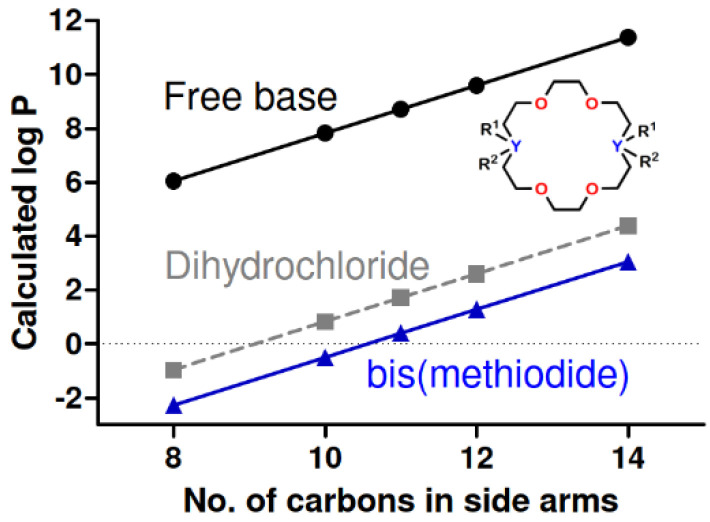
Calculated log P octanol–water partition coefficients for C_8_, C_10_, C_11_, C_12_, and C_14_ lariat ethers. For the free bases, R^1^ = H, R^2^ is absent and Y = N. For the dihydrochlorides, R^1^ = R^2^ = H and Y = N^+^. For the methiodides, R^1^ = H, R^2^ = CH_3_, and Y = N^+^.

An additional solubility property is evaluated as the partition coefficient between water and *n*-octanol. Figure 3 shows calculated log P values for the C_8_-C_14_ lariats and their salts. The data were calculated using the log P partitioning software in the ChemAxon Marvin Sketch program. As expected, the graph shows linear increases in log P as the number of carbons in the sidearms increased. Of course, these calculations reflect a neutral water/*n*-octanol system and not media-containing cations, sugars, protein, or buffers. The perfect linearity results from the computational model rather than experiment.

The difference in the solubilities of C_10_LE•2MeI (**3**) and C_12_LE•2MeI (**6**) observed experimentally (Figure 2) was unexpected. The solubilities of the various lariat ethers seem to be similar or identical in the two media except for C_12_LE•2MeI (**6**). It is tempting to suggest that because Mueller Hinton broth contains such elements as beef extract and starch, differences might be expected compared to phosphate-buffered saline. If an interaction occurs with the lariat ether methiodides, it should be manifested by both C_10_LE•2MeI (**3**) and C_12_LE•2MeI (**6**). Although the effect was less dramatic for C_10_LE•2MeI (**3**) than for C_12_LE•2MeI (**6**), **3** and **6** were the only compounds in this series to show such a deviation. It seems likely that the organic elements of the MH broth as well as sidearm disposition (see above) played a role in the difference, but such an explanation remains speculative.

### 2.4. Minimum Inhibitory Concentration (MIC) Studies

MICs were determined [46] for LEs and antibiotics against *E. coli* K12, *S. aureus* 1199B, and tet^R^ *E. coli*. The K12 strain was benign, but more clinically relevant than DH5α. The strain of *E. coli* designated tet^R^ was developed in our laboratory and incorporates the tetracycline efflux pump [50]. The results are shown in Table 3 below. The MICs of norfloxacin and tetracycline were high as expected because they were determined against resistant strains of bacteria. The MICs of C_10_LE (**1**) and C_12_LE (**4**) against *E. coli* were similar to the previously reported values. Note that the lariat ether free bases having *n*-octyl, *n*-tetradecyl, or *n*-hexadecyl sidearms all showed no activity against K12 *E. coli* at concentrations lower than 128 µM.

Compounds **1**–**6** were all more active against Gram-positive *S. aureus* than Gram-negative *E. coli*. The MICs of C_10_LE (**1**) and C_10_LE•2HCl (**2**) were similar against the three bacterial strains tested, although potency was slightly higher against Gram-positive *S. aureus*. It is interesting to note that the difference in solubility between **1** and **2** did not significantly affect the MICs, which ranged only from 2–10 µM. The MIC of C_12_LE (**4**) against *S. aureus* (32 µM) was several times higher (less potent) than that of C_10_LE (**1**). This comports with the sodium release data previously published: **4** was a less active ion transporter in a membrane than compound **1** [48]. The antimicrobial activities of the HCl and MeI salts of C_12_LE (**5** and **6**) against *S. aureus* were greater than those of the free bases. C_12_LE•2MeI (**6**) showed the greatest potency (MIC = 1 µM) against *S. aureus*. Against Gram-negative tet^R^ *E. coli*, C_12_LE•MeI, **6**, was active (MIC = 16 µM), but C_12_LE free base **4** and methiodide **5** were inactive. Against both *E. coli* and *S. aureus*, the C_10_LE free base was more active than C_12_LE (**4**). The C_12_LE•2HCl (**5**) salt was more active than C_10_LE•2MeI (**6**).

The LEs may disrupt ion homeostasis and/or alter membrane permeability. Changes in membrane permeability may enhance antimicrobial influx. Altered permeability may also permit unregulated flow of cations into and out of the organism. To the extent the latter occurs, the function of any enzyme that requires ion regulation will be compromised. We have shown that the hydraphile relatives of LEs are potent efflux pump inhibitors [45]. It should also be noted that the presence of positive charges in **2**, **3**, **5**, and **6** should enhance selectivity for the negative surfaces of bacteria compared to mammalian cells.

### 2.5. Membrane Permeability

**Figure 4:**
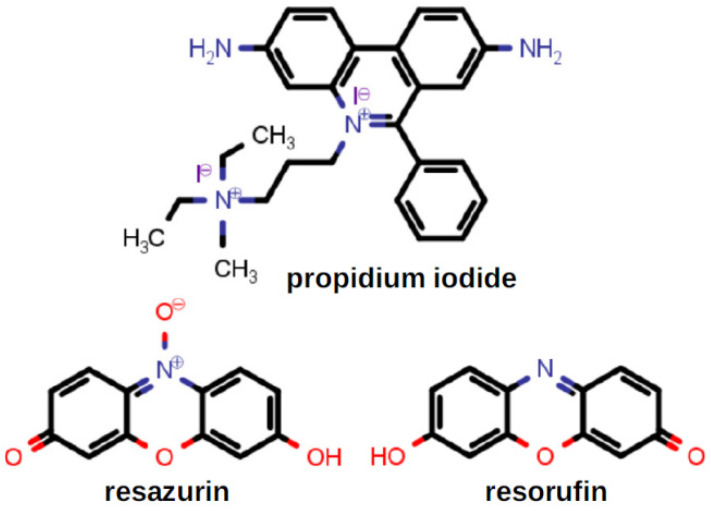
The chemical structures of propidium iodide and the resazurin–resorufin pair.

Membrane permeability was assessed by using the dyes propidium iodide and alamar blue. Their structures are shown in Figure 4. Propidium iodide (PI) is a fluorescent dye that normally does not penetrate intact biological membranes. However, when PI does enter a cell, it intercalates within the DNA structure. Intercalation of PI does not appear to favor any particular nucleobase sequence. When intercalation occurs, a strong fluorescent emission can be detected. Alamar blue, based on resazurin, is a nontoxic weakly fluorescent dye that is sensitive to reduction potential within vital cells. When reduced, it is converted into highly fluorescent resorufin (7-hydroxy-3H-phenoxazin-3-one). The reduction occurs during respiration in vital cells; formation of highly fluorescent resorufin demonstrates active metabolism and therefore cellular vitality.

**Figure 5:**
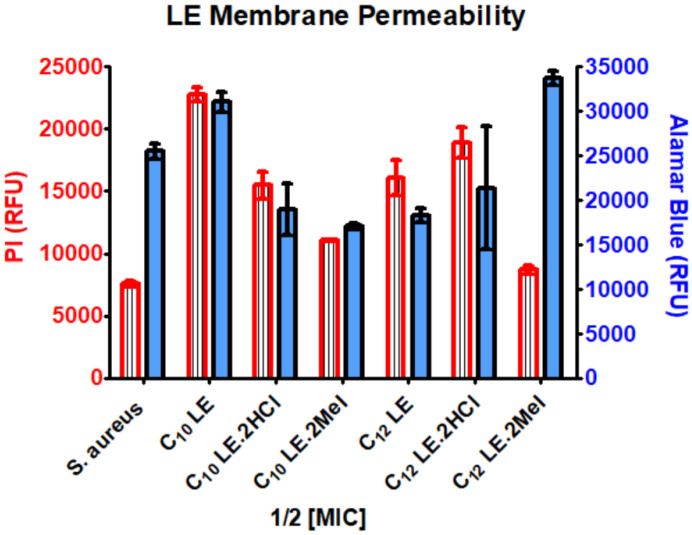
Assay of membrane permeability and vitality of *S. aureus* in the presence of **1**–**6** administered at ½ MIC. Increase in PI fluorescence (red striped bar)/membrane penetration into *S. aureus*. The blue bars represent survival assayed by the alamar blue test. Error bars represent the standard deviation in three independent trials.

**Figure 6:**
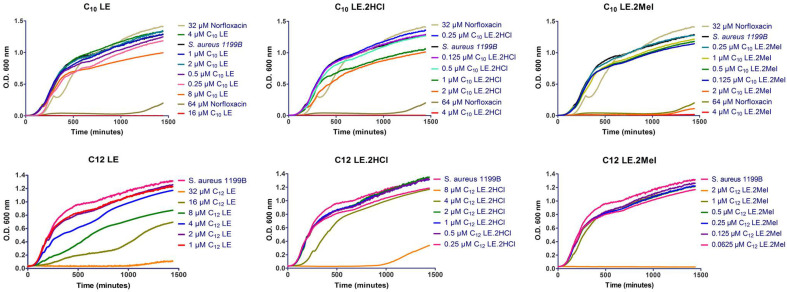
Growth curves for *S. aureus* 1199B in the presence of norfloxacin and compounds **1**–**6**. Optical density (600 nm) was measured every 4 min for 24 h (1440 min) using a multimodal plate reader. The data shown are the averages of three trials in each case. Error bars have been omitted for clarity.

The combination of alamar blue and propidium iodide allowed us to assess the membrane permeability of *S. aureus* while confirming cellular vitality. Results of the PI/alamar blue studies with *S. aureus* are summarized in the graph of Figure 5. When *S. aureus* (1199B) was exposed to the two dyes, penetration of PI was relatively low and cellular vitality remained high. Compounds **1**–**6**, were administered at ½ MIC, at which concentrations toxicity was not expected (see growth curves below in Figure 6). In general, cellular penetration and vitality correlated. The obvious exception was C_12_LE•MeI (**6**), which showed a level of PI penetration similar to that observed for *S. aureus* alone. The latter is a particularly interesting observation because **6** showed the highest potency against *S. aureus*, matched only by C_10_LE•MeI (**3**), which did show good penetration. These results suggest that potency cannot be explained solely by altered membrane permeability.

Another potential variable in the assessment of potencies of **1**–**6** against *S. aureus* is the environment in which the study is conducted. It is clear, for example, that the conversion of **1** → **2** and **4** → **5** will be favored at lower pH. Modest changes in pH are unlikely to alter the MIC values for methiodides **3** and **6**. Indeed, changes in MIC were observed for **1**, **2**, **4**, and **5** as the pH was increased from 6.4 to 8.4, while the MICs for **3** and **6** were unchanged. These studies were conducted in Mueller Hinton (MH) microbiological growth medium. Both MH versions are more complex media than phosphate-buffered saline (PBS). MH typically contains beef extract, casein hydrolysate, starch, and water. Once the components are mixed, the pH is adjusted to neutral. MHII, used here, also contains Ca^2+^ and Mg^2+^ ions. The results are shown in Table 4.

### 2.6. Growth Curves

In the studies above, **1**–**6** were administered at ½ MIC concentrations. At these concentrations, no toxic effect of the compound on cells was anticipated. In order to confirm the effect of compound concentration on *S. aureus* viability, growth curves were conducted for each compound. In all cases, *S. aureus* alone was used as control. For the three C_10_LE compounds (**1**–**3**), 32 µM and 64 µM concentrations of norfloxacin were added to the norfloxacin-resistant *S. aureus* strain as an additional control. The concentration ranges for each of compounds **1**–**6** varied with their potencies.

In all cases, bacterial growth was unaffected at ½ MIC concentrations of the lariat ether or lariat ether derivative. In general, the data show that against *S. aureus* 1199B, which is norfloxacin-resistant, there was little response to low concentrations of the LEs. The single exception is C_12_LE (**4**). While the MIC against this bacterium was 32 μM, growth effects were observed at ½ MIC and lower. This was the only LE among the six shown for which such behavior was observed. It is especially surprising because the behavior of C_10_LE (**1**) and C_12_LE (**4**) usually correlate. At present, the reason for this apparent anomaly is unclear.

### 2.7. Combination Studies

The focus on bacterial growth at ½ MIC of **1**–**6** derives from the potential of these compounds to function as adjuvants. In combination with a known drug, an adjuvant has the potential to enhance its potency. In some cases, the adjuvant may thwart a resistance mechanism. An example is the combination antibiotic Augmentin^®^, which combines amoxicillin with clavulanic acid: the latter component inhibits degradation of the penicillin by β-lactamase enzymes. Recent studies have shown that colistin, a membrane penetrating antibiotic, can be used with a potency increasing adjuvant [58].

**Figure 7:**
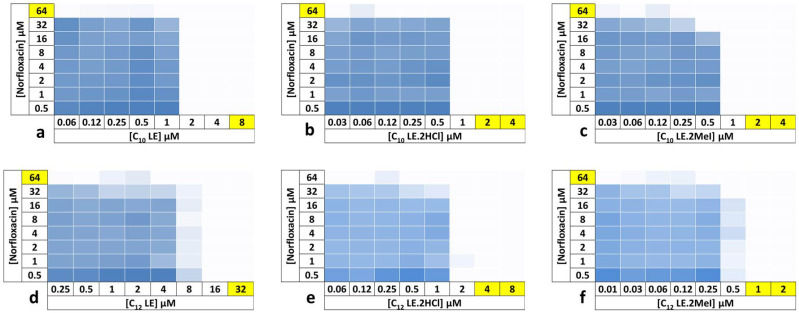
Checkerboard experiments with **1**–**6** in combination with norfloxacin against *S. aureus* 1199B. The results are the average of three trials. Data represent the average inhibition of growth for three trials. Shades of blue color indicate growth whereas a white color indicates inhibition of growth > 90%. (**a**) C_10_LE, **1**; (**b**) C_10_LE,2HCl, **2**; (**c**) C_10_LE.2MeI, **3**; (**d**) C_12_LE, **4**; (**e**) C_12_LE.2HCl, **5**; (**f**) C_12_LE.2MeI, **6**.

Each of the checkerboard analyses shown in Figure 7 combines one of **1**–**6** with norfloxacin. *S. aureus* 1199B is resistant to norfloxacin. By combining varying concentrations of both norfloxacin and a lariat ether or lariat ether derivative, potency enhancement, if any, can be determined. In the graphs (checkerboards) shown, the potency of each concentration combination can be assessed visually. The darkest shades in each graph correspond to the least potent combinations. The absence of color shows 90% or greater efficacy of the adjuvant with norfloxacin against the norfloxacin-resistant organism.

An example is the combination of C_10_LE (**1**) and norfloxacin, as shown in the upper left panel of Figure 7. *S. aureus* was resistant to norfloxacin at up to 32 µM, but succumbed at 64 µM. However, when **1** is co-administered at 2 µM, only 0.5 µM norfloxacin was required to effect 90% growth inhibition. This was a 128-fold increase in the potency of norfloxacin against this resistant organism.

In the figure above, the MICs for norfloxacin and the LEs are highlighted. Where two values are highlighted, the MIC lies between the two, leading to a range of potentiation values. In all cases, however, the enhancement of potency was significant for compounds **1**–**6**, regardless of charge or alkylation.

### 2.8. Complexation of Antimicrobials by Lariat Ethers

**Figure 8:**
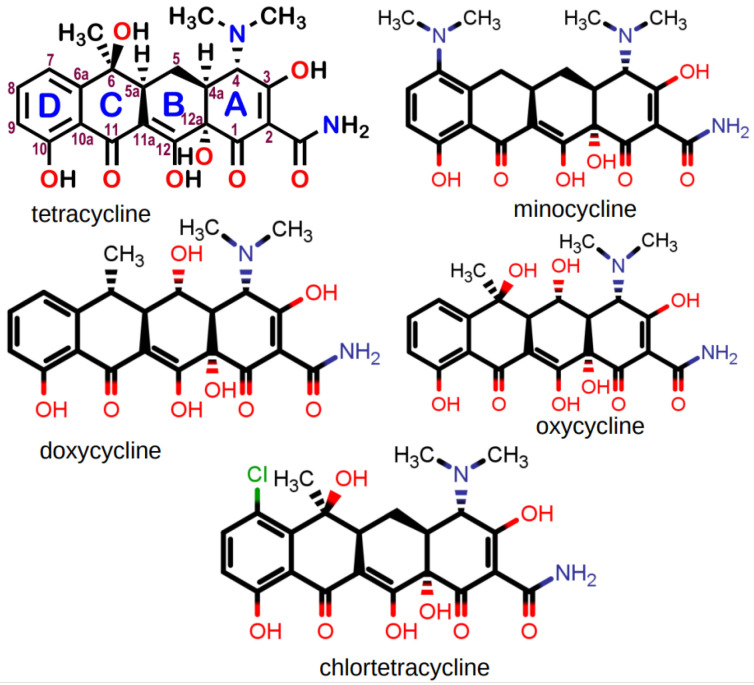
Chemical structures of five tetracycline compounds studied as their hydrochloride salts.

We recently reported [59] that the presence of C_10_LE enhanced the penetration of tetracycline hydrochloride in a model membrane system. Evidence for an interaction between the macrocycle and antimicrobial salt was obtained by showing that dichloromethane insoluble tetracycline hydrochloride dissolved on a 1:1 basis with the lariat ether. The dissolution was observed visually in CH_2_Cl_2_ solution and quantitated by NMR analysis in CD_2_Cl_2_. In the visualization experiment, the insolubility of the antimicrobial salt was demonstrated in either CH_2_Cl_2_ or CHCl_3_ (with identical results). The solubility of the lariat ether alone was likewise confirmed. A combination of the lariat ether, the antimicrobial salt, and solvent were mixed. Dissolution of the salt provided visual confirmation of the complexation, presumably by a supramolecular interaction. In the NMR experiments, excess salt was used and the solution filtered before obtaining the spectrum. Figure 8 shows the structures of the five tetracycline derivatives studied.

The complexation studies were undertaken in several ways. In previous work [60], we showed by neutron reflectance that the presence of C_10_LE enhanced the penetration of tetracycline in a model membrane. In order to obtain additional information about tetracycline’s behavior, we attempted to dissolve its hydrochloride salt in CH_2_Cl_2_ to no avail. The solvent was chosen because its dielectric constant mimicked that of *n*-octanol, a common membrane model. C_10_LE was freely soluble in CH_2_Cl_2_. A solution of C_10_LE was exposed to powdered tetracycline hydrochloride, mixed, and filtered. A clear, but slightly yellow solution was obtained showing that the drug had fully dissolved.

**Figure 9:**
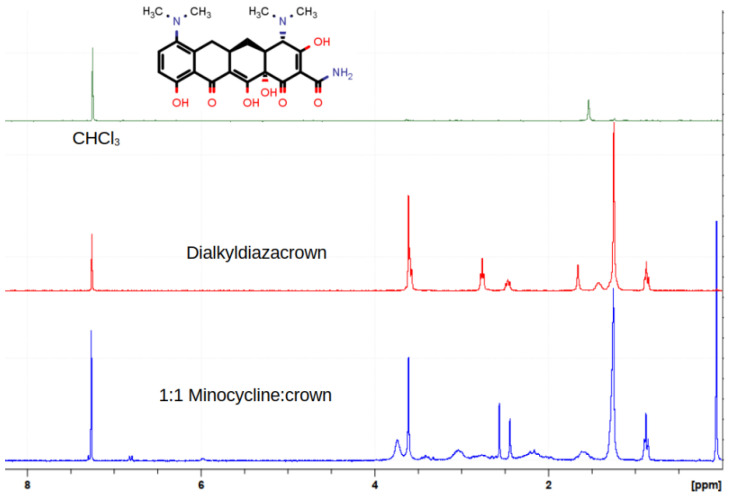
NMR stack plot showing the NMR spectrum of minocycline hydrochloride in CDCl_3_ (**top**), C_10_LE in CDCl_3_ (**center**), and both crown and drug dissolved in CDCl_3_ in a 1:1 ratio (within experimental error).

In order to quantitate the dissolution/complexation phenomenon, a similar procedure was conducted using either CD_2_Cl_2_ or CDCl_3_ and the complex stoichiometry was determined by evaluating NMR integrals. Each NMR experiment was conducted at least in triplicate. A representative stack plot is shown for minocycline hydrochloride in Figure 9. An effort to obtain additional complexation data by using infrared techniques was unsuccessful.

Similar NMR experiments were conducted with the tetracycline series of compounds. The results are recorded in Table 5.

**Figure 10:**
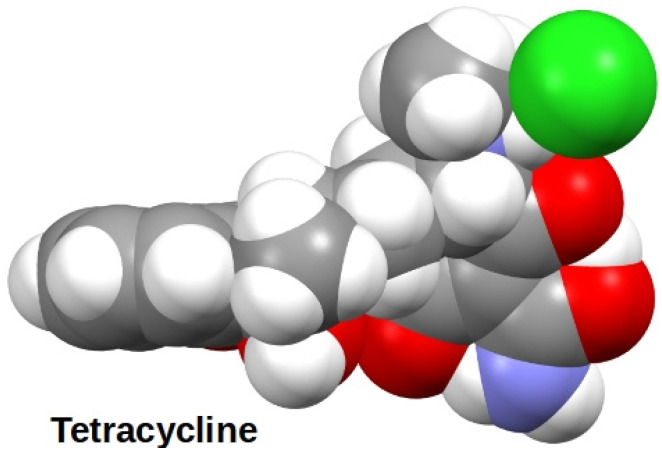
Solid state structure of tetracycline hydrochloride rendered by Mercury software from CCSD XAYCAB.

An examination of the numerous NMR experiments showed that the most prominent changes in the spectra occurred in the signals proximate to the macro ring nitrogen atoms. The inference drawn from this was that hydrogen bonding facilitated by tetracycline’s hydrochloride was providing a primary link between drug and crown. It should be noted that the tetracycline family of molecules, although represented in line-angle drawings as four fused linear rings, actually are bent. Figure 10 shows the structure of tetracycline hydrochloride rendered in the space-filling representation from the CCSD structure (XAYCAB) [61] using Mercury software v15.6.29.0.

**Figure 11:**
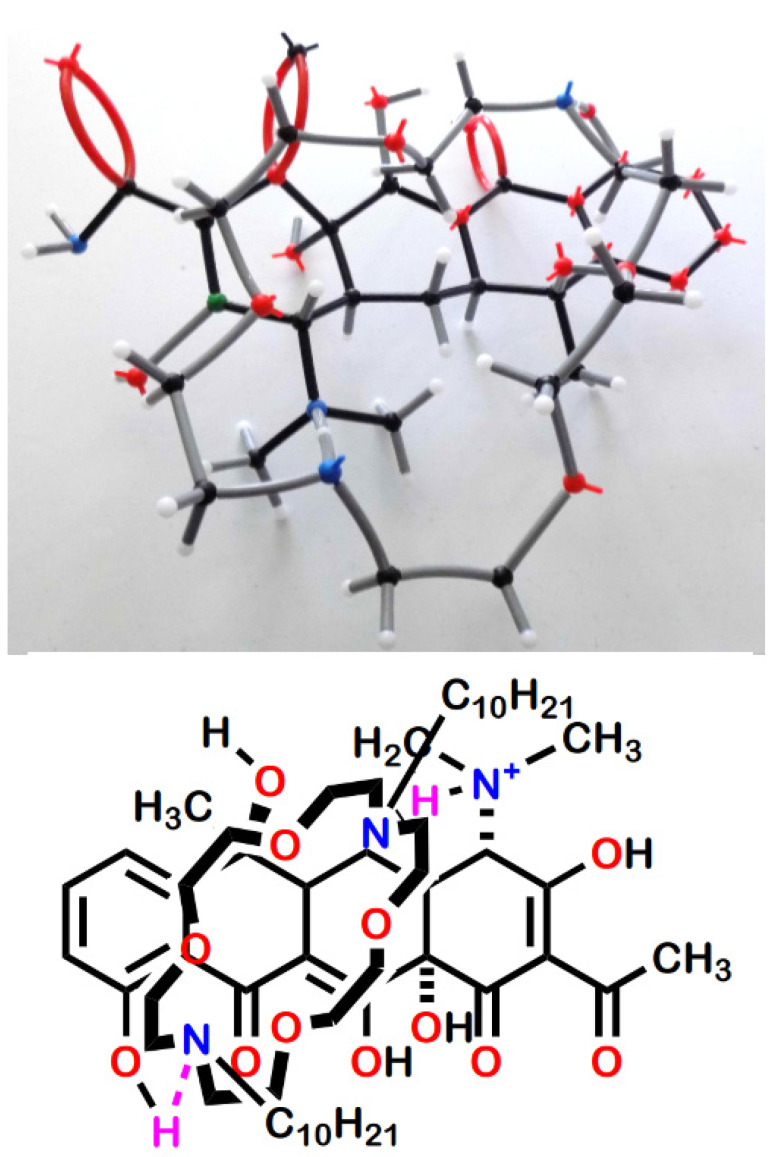
Proposed H-bonding interactions that stabilize complexation in the tetracycline series.

An examination of various types of molecular models suggested the contacts illustrated in Figure 11. The top panel shows the association using framework models. An attempt was made to illustrate the postulated complex using CPK space-filling models, but the possible bonding pattern was obscure in this representation. The suggested arrangement is shown in wireframe models and the line-angle drawing in the lower panel of Figure 11. The two presumed hydrogen bonds are shown in the drawing by using purple hydrogen atoms. The two hydrogen bonds are donated from tetracycline to the macrocycle. The protonated dimethylamino group at position 4 in the A ring of tetracycline donates to one macro ring nitrogen atom and the phenolic hydroxyl at position 10 in the D ring provides the other hydrogen.

The lack of any complexation interaction between C_10_LE (**1**) and chlortetracycline was surprising. The explanation did not seem to lie in a difference in pKa values or significant changes in polarity compared to tetracycline. However, an examination of models suggested that the chlorine in the 7-position of the D ring presented a steric impediment to the macrocycle accessing the phenolic hydroxyl in the D-ring. The alternate hydroxy groups are either tertiary and hindered (positions 6 and 12a) or enolic and part of a β-diketone system (position 12, see Figure 8).

As expected, tetracycline hydrochloride was freely soluble in water whereas C_10_LE (**1**) is only slightly soluble. In an inversion of the experiments described above, the water-insoluble diazacrown was drawn into aqueous solution on a 1:1 basis. Because of water’s solvating power and the inferred stabilization of the complex by H-bonding, this result was unexpected. As in the other complexation studies, the result was confirmed in triplicate. Quantitation was obtained by NMR studies conducted in D_2_O.

### 2.9. Dynamic Light Scattering (DLS)

**Figure 12:**
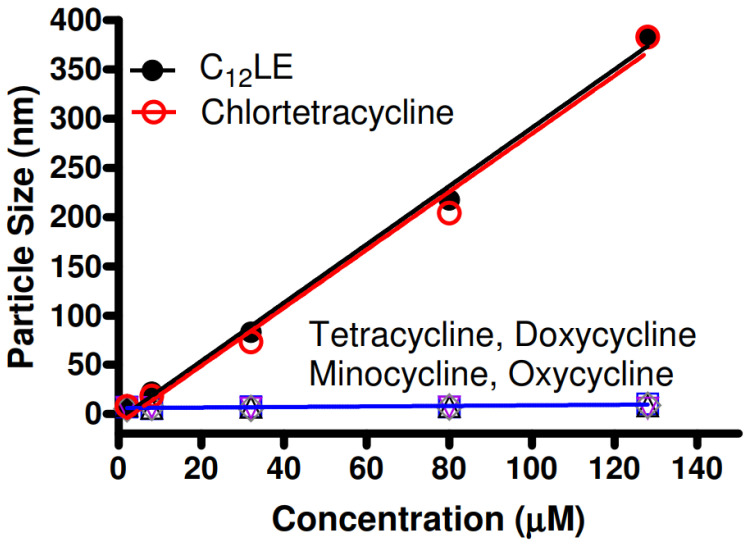
Dynamic light scattering data in water for C_12_LE (**4**, filled circles) and tetracycline (open squares), doxycycline (open diamonds), minocycline (open triangles), oxycycline (open inverted triangles), and chlortetracycline (open circles) all as hydrochlorides.

It is known that dialkyldiazacrowns form stable aggregates in aqueous solution [57,62]. It was hypothesized that complexation between antimicrobials and macrocycles would alter or prevent the ability of the complex to form aggregates. The graph of Figure 12 shows the results of dynamic light scattering for C_12_LE (**4**) in water along with the combinations of the crown and the five tetracycline derivatives. Of the five tetracyclines studied, only chlortetracycline hydrochloride failed to complex with C_12_LE (**4**). The graph clearly shows that aggregation was completely impeded by all of the tetracyclines except for chlortetracycline hydrochloride. Indeed, the particle sizes observed when chlortetracycline is present was almost indistinguishable from particle formation by C_12_LE (**4**) alone.

**Figure 13:**
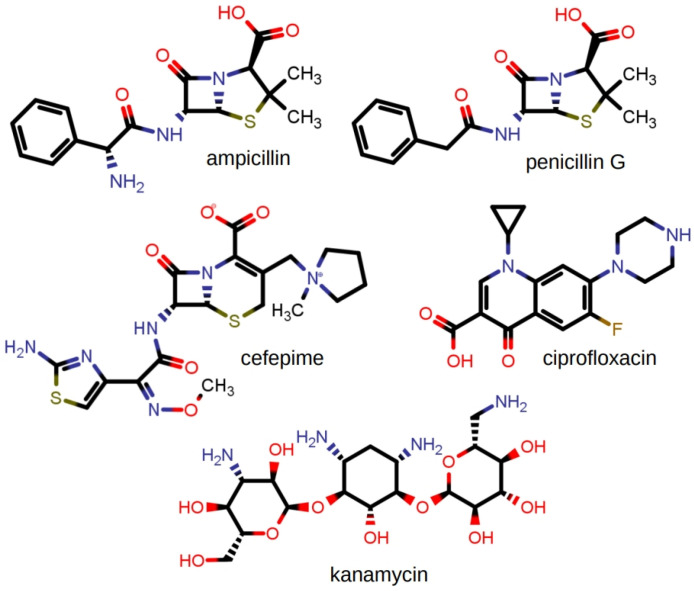
Chemical structures of ampicillin, penicillin G, cefepime, ciprofloxacin, and kanamycin. Dynamic light scattering and complexation data are shown in Figure 14.

The studies described for the tetracycline family were extended to a number of antimicrobials. The group included ampicillin, penicillin G, cefepime, ciprofloxacin, and kanamycin. This group does not comprise an analogous series as was possible with the group of tetracycline derivatives. Further, the tetracycline derivatives were all hydrochloride salts. In the present group, the structures of which are shown in Figure 13, only ciprofloxacin was obtained as the hydrochloride salt. The other compounds were examined in the forms available as commercial pharmaceuticals. Ampicillin was used as the trihydrate, penicillin G as the sodium salt, cefepime was used as the betaine, and kanamycin (primarily A but containing some B and C) was used as the monosulfate salt.

The group of compounds illustrated in Figure 13 is too disparate both structurally and in charge states for any direct comparison to be made among them. In the presence of C_12_LE (**4**), neither cefepime nor kanamycin was drawn into CHCl_3_ or CDCl_3_ solution. The complexation ratios for the other three antimicrobials were (drug:C_12_LE) ciprofloxacin, 2.8; ampicillin, 5.1; and penicillin G (benzylpenicillin), 7.1. As with the tetracyclines, all values represent the average of at least three separate experiments.

Because of the differences among these compounds, it is unreasonable to expect any single binding mechanism to account for the interactions. It is therefore not surprising that there was modest correlation at best between the complexation ratios and the dynamic light scattering data. If these antimicrobials were directly comparable, an order for aggregation might be expected according to the complexation data. This order of particle sizes would hypothetically be ciprofloxacin < ampicillin < penicillin G and the particle sizes for cefepime and kanamycin would be largest and similar in size. The largest particles are, indeed, found for cefepime and kanamycin, which exhibit no complexation with C_12_LE (**4**). The presence of ampicillin, penicillin G, and ciprofloxacin each diminished the aggregation of C_12_LE (**4**), although not in the order that might be anticipated from the complexation data.

**Figure 14:**
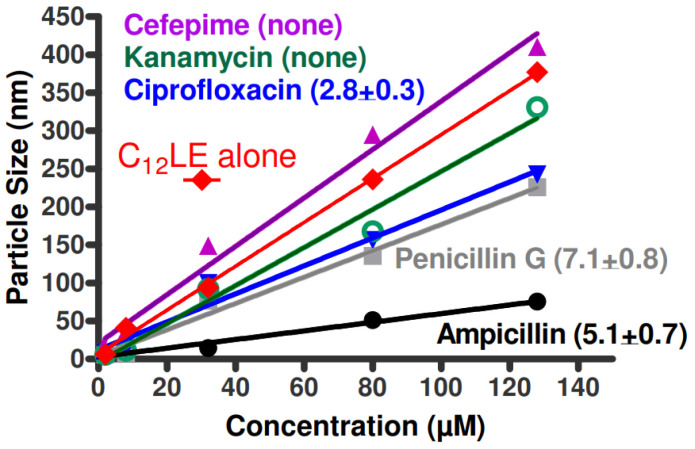
Dynamic light scattering (particle sizing) and complexation data for the C_12_LE and the group of compounds for which the structures are shown in Figure 13. The NMR-determined complexation data are shown in parentheses.

Neither cefepime nor kanamycin showed complexation with C_12_LE (**4**) when studied in the NMR extraction experiment. The lack of interaction was confirmed by the fact that C_12_LE aggregation showed the highest slopes: 3.2 for cefepime and 2.5 for kanamycin. The average of these was 2.85, which differed from the slope of C_12_LE (**4**) alone of 2.95 (not shown on Figure 14). For the remaining three compounds, ampicillin, penicillin, and ciprofloxacin, the shallower slopes indicated an effect on aggregation that diminished the ability of C_12_LE (**4**) to form large particles.

Ciprofloxacin was studied as the hydrochloride monohydrate. An interaction between the protonated secondary nitrogen with the neutral diazacrown seemed possible. The complexation ratio of 2.8 (crown:drug) showed that an interaction occurred, and its apparent weakness was reflected in the 1.8 slope, which was about 60% of the particle size profile of C_12_LE (**4**) alone.

Ampicillin and penicillin G are quite similar structurally. However, penicillin G was obtained as the sodium salt and ampicillin as the trihydrate. Both compounds have two amidic nitrogen atoms and a carboxylate group. The acidities (pKa) of the carboxyl groups are generally in the 2.6–2.8 range. Ampicillin has an additional free amino group as part of the phenylglycine side chain. It was obtained as the trihydrate and was assumed to be in the betaine form. The diazamacrocycle of C_12_LE (**4**) can bind the sodium cation present in penicillin G. Likewise, the crown can bind the ammonium salt if ampicillin is present in the betaine form. Both the NMR and DLS evidence confirmed an interaction of C_12_LE (**4**) with both, albeit to different extents. Indeed, the interactions may have occurred owing to entirely different interactions, which have not been determined in the present work. The fact that ampicillin shows a lower crown:drug ratio and stronger hindrance to aggregation than penicillin G supports a different mechanism of binding.

Our current understanding of the interaction of the various antimicrobials with lariat ethers involves hydrogen bonding. If that explanation obtains, it is somewhat surprising that complexation with the tetracyclines was observed in D_2_O as well as in CDCl_3_. Further, all of the DLS experiments were conducted in water and interactions were apparent in that assay as well. The fact that complexation occurred in water bodes well for the potential application as an adjuvant for antimicrobial therapy.

### 2.10. Membrane Depolarization

Our hypothesis is that the lariat ethers, to a greater or lesser extent, penetrate bacterial membranes. If so, there are at least two obvious consequences. First, the exogenous amphiphile may disrupt the membrane’s local structure and cause an increase in permeability. To the extent that this happens, more antibiotic may diffuse into the bacterial cytosol. Second, any change in membrane permeability or integrity should affect ion homeostasis. A change in the membrane polarization of the organism owing to deregulation of proton or potassium cation concentrations should also be detectable.

**Figure 15:**
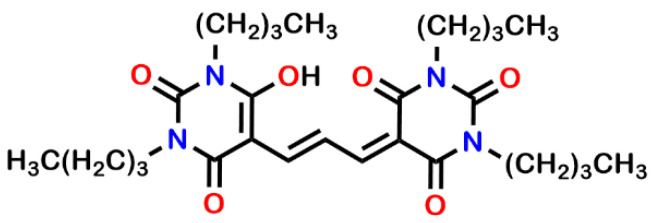
Chemical structure of DiBAC4(3) (bis-(1,3-dibutylbarbituric acid)trimethine oxonol).

Disruption of ion homeostasis by **1**–**6** should be apparent in the associated membrane depolarization. The membrane dye, DiBAC4(3) (bis-(1,3-dibutylbarbituric acid)trimethine oxonol), shown in Figure 15, will exhibit different fluorescent signals in accordance with polarization or depolarization [63]. The dye is readily absorbed by bacteria owing to the negative internal potential [64]. Disruption of ion homeostasis will affect both proton and potassium cation regulation [65]. The internalized, self-quenched dye will be released and increased fluorescence will be observed.

**Figure 16:**
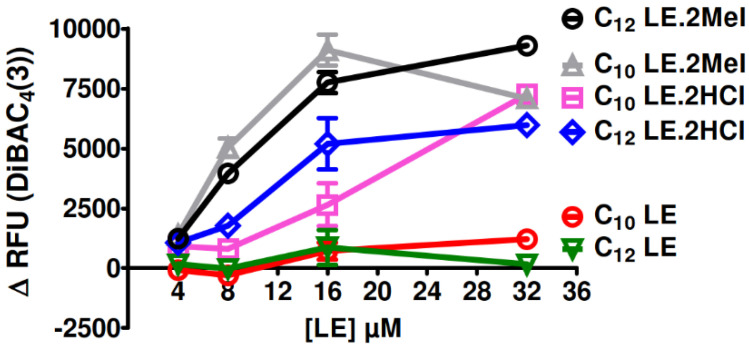
Concentration dependence of membrane depolarization determined for **1**–**6** by DiBAC4(3) dequenched fluorescence.

Two types of experiments were performed to assess membrane depolarization. Figure 16 shows the changes in fluorescence observed when *S. aureus* was exposed to DiBAC4(3) and then to varying concentrations of **1**–**6**. If ion homeostasis is affected by the presence of any of these compounds, the fluorescent response should increase as the lariat ether concentration increased. The graph of Figure 16 shows increased fluorescence generally up to concentrations of 16 µM for **1**–**6**, with little change thereafter.

**Figure 17:**
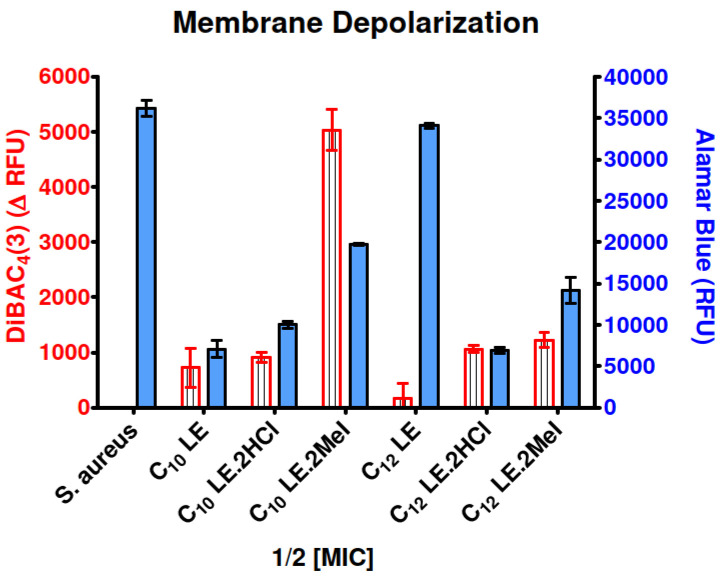
Increase in membrane depolarization of *S. aureus* in the presence of **1**–**6** at ½ MIC assessed by DiBAC_4_(3) fluorescence. Error bars represents standard deviation in three independent trials.

A second series of experiments was performed to determine the relationship of disrupted ion homeostasis, as reflected in membrane depolarization [65] with cellular vitality (Figure 17). In this case, *S. aureus* was confronted by each of **1**–**6** at ½ MIC. Alamar blue (see above) was used to determine cell viability [66]. No depolarization was apparent in the *S. aureus* control in the absence of a lariat ether or lariat ether derivative. Depolarization was greatest for C_10_LE•MeI (**3**). Similar levels of depolarization were observed for **1**, **2**, **5**, and **6**. Only C_12_LE (**4**) failed to show any significant evidence of membrane depolarization. As shown in Table 3 (above), **4** showed the lowest potency (highest MIC) against *S. aureus*. We note that the MIC values for *S. aureus* observed for **2**, **3**, **5**, and **6** were similar.

### 2.11. Planar Bilayer Voltage Clamp Study

**Figure 18:**
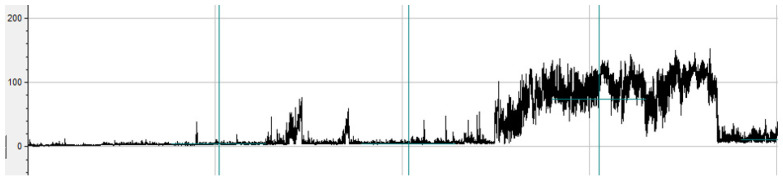
Planar bilayer voltage clamp trace for C_11_LE•2MeI (**8**) at 70 mV. Axes: Y = millivolts and X axis = 10 s per section. Conditions: soybean asolectin membrane, KCl (450 mm) buffer solution (HEPES, 10 mm, pH 7).

In a previous study, *N,N*′-diundecyl-4,13-diaza-18-crown-6 was found to form stable channels in planar asolectin membranes [67]. This compound was therefore converted into its *bis*(methiodide) to form C_11_LE•2MeI (**8**). Compound **8** was analyzed, as was the free base, and the trace shown in Figure 18 was obtained. It is clear from the trace shown that only spiking behavior was observed and a large opening eventually emerged. Although this trace confirms membrane insertion, it is clear that at least this methiodide does not form stable channels. The formation of a large opening in the membrane comports with the expectation of increased permeability. Such an opening is also in accord with the observation (see above) of depolarization of bacterial membranes.

### 2.12. Efflux Pump Inhibition

**Figure 19:**
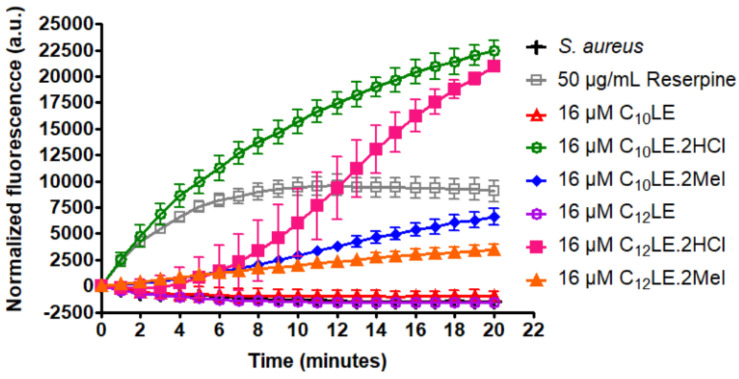
Inhibition of NorA efflux pumps in *S. aureus* 1199B in the presence of compounds **1**–**6**. Increase in EthBr fluorescence observed by its accumulation in *S. aureus* 1199B cytosol in the presence of 16 µM compounds **1**–**6**. Error bars represents standard deviation in three independent trials.

An important reason for the choice of *S. aureus* 1199B for study is that its resistance to norfloxacin is known to result from the presence of the Nor A efflux pump [50,68]. Efflux pumps require regulated ion balance to function so that exogeneous substances such as antibacterials can effectively be ejected. Ethidium bromide (EthBr) can penetrate bacteria and is detectable by its fluorescence. To the extent that ion homeostasis is disrupted, ion balance is deregulated, and efflux pump function is compromised or inhibited. Figure 19 shows the results of a comparative study conducted at a concentration of each lariat ether of 16 µM. *S. aureus* is one control and reserpine, administered at 50 µg/mL is the second. Reserpine is a typical efflux pump inhibitor and shows a potency similar to that of C_10_LE•MeI (**3**). The concentration of reserpine at 50 µg/mL corresponds to 80 µM. Thus, **3** is approximately five times more potent as an efflux pump inhibitor than reserpine.

In this study, the two lariat ether hydrochlorides, **2** and **4**, showed the most potent efflux pump inhibition. This study used concentrations that were identical for each of the lariat ethers rather than their MIC or ½ MIC values. The graph is intended to show efflux pump inhibition at concentrations that can be directly compared. Of course, efflux pump inhibition is only one of several possible influences on the potency of the lariat ethers functioning either as drugs per se or as adjuvants.

### 2.13. Cytotoxicity of ***1***–***6***

**Figure 20:**
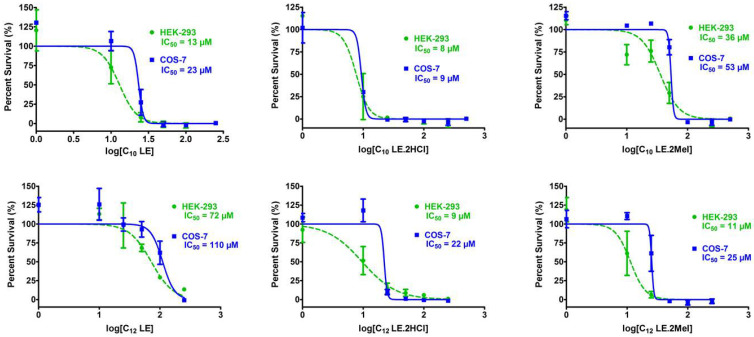
Cytotoxicity of compounds **1**–**6** against HEK-293 and COS-7 cell lines. In each graph, the abscissa represents concentrations of **1**–**6** on a logarithmic scale and the ordinate shows the survival of mammalian cells (percentage survival). Error bars represents standard deviation in three independent trials and IC_50_ were calculated in each case.

The potential toxicity of **1**–**6** is a critical factor in their application either as an antimicrobial or an adjuvant. In vitro toxicity studies were therefore conducted using human embryonic kidney (HEK-293) and simian kidney (COS-7) cells. The graphs shown in Figure 20 show the percentage survival of each cell type as a function of lariat ether concentration on the logarithmic x-axis. In each graph, the IC_50_ value is specified for each compound and cell type. The value of IC_50_ is the concentration of lariat ether that inhibits the growth of 50% of the cells studied.

In general, the most potent compounds were **1**, **2**, **3**, and **6**. The IC_50_ values for **1**, **2**, and **6** against either HEK-293 were less than or equal about 10 µM, making them poor candidates for use as drugs. However, the IC_50_ for methiodide salt **3** was 36 µM, giving it some margin of utility. Considering that the compound would be dosed at ½ MIC when used as an adjuvant, **3** holds potential in that role. Compound **4** showed low toxicity, but it was essentially inactive against the *E. coli* strains tested. Its activity against *S. aureus* was only 32 µM.

## 3. Materials and Methods

### 3.1. Compound Preparation

^1^H NMR were recorded at 400 MHz in CDCl_3_ and are reported in ppm (delta) downfield from internal Me_4_Si. Abbreviations used in NMR: bs = broad singlet, *pseudo*-d = *pseudo*-doublet, m = multiplet. Melting points were determined on a Thomas–Hoover apparatus in open capillaries and are uncorrected. All reactions were conducted under dry argon unless otherwise noted. All reagents were the best grade commercially available and were dried, distilled, and/or recrystallized as appropriate, prior to use.

***N,N*′-Di-*n*-decyl-4-13-diaza-18-crown-6, 1** was prepared as reported in reference [48].

***N,N*′-Di-*n*-decyl-4-13-diaza-18-crown-6 dihydrochloride, 2**, was prepared from **4** (200 mg) dissolved in dioxane (5 mL). A solution (commercial) of HCl in dioxane was (0.8 mL) was added and the mixture stirred for 5 min. The solvent was evaporated, washed with diethyl ether, and filtered. The resulting solid, obtained in 66% yield, had a MP 135–138 °C. ^1^H-NMR: 0.92 (6H, t); 1.29–1.35 (28H, m); 1.85 (4H, bs); 3.2 (4h, bs); 3.42, 3.51 (8H, *pseudo*-d); 3.70 (8H, m); 3.97 (4H, bs); 4.12 (4h, bs). 

***N,N*′-Di-*n*-decyl-4-13-diaza-18-crown-6 bis(methiodide), 3.** The free base, **1** (50 mg) was dissolved in toluene (5 mL) and 60 µL of CH_3_I was added under argon. The mixture was stirred for 16 h, after which the solvent was evaporated. Methanol (5 mL) was added followed by diethyl ether, which was added dropwise to force precipitation. The solid thus obtained was washed with cold hexane to yield **3** in 53% yield as a colorless solid MP 174–176 °C. 1H-NMR: 0.88 (6H, t); 1.26–1.36 (28H, *pseudo*-d); 1.73 (4H, bs); 3.35–4.11 (34H, m). 

***N,N*′-Di-*n*-dodecyl-4-13-diaza-18-crown-6, 4** was prepared as reported in [48].

***N,N*′-Di-*n*-dodecyl-4-13-diaza-18-crown-6 dihydrochloride, 5** was prepared from **4** (200 mg) dissolved in dioxane (5 mL). A solution (commercial) of HCl in dioxane (0.8 mL) was added and the mixture stirred for 5 min. The solvent was evaporated, washed with diethyl ether, and filtered. The resulting solid, obtained in 82% yield, had a MP 141–144 °C. ^1^H-NMR: 0.9 (6H, t); 1.27–1.34 (36H, m); 1.70–1.84 (6H, m); 3.17–4.10 (28H, m). 

***N,N*′-Di-*n*-dodecyl-4-13-diaza-18-crown-6 bis(methiodide), 6,** was prepared in analogy to **3**, using 300 mg in 5 mL of toluene. Methyl iodide (0.31 mL) was added and the mixture stirred under argon for 3 h. The solvent was evaporated and the residue dissolved in minimum methanol. Dropwise addition of ether led to a white solid (240 mg, 54%) melting at 186–189 °C.

***N,N*′-Di-*n*-undecyl-4,13-diaza-18-crown-6, 7**. The preparation of this compound is reported in [44].

### 3.2. Minimum Inhibitory Concentrations

All bacteria were grown in Mueller Hinton II (MHII) media. The cells were grown overnight from one colony forming unit (CFU) in 2 mL media. On the day of the experiment, the bacteria were knocked back to OD_600_ = 0.100 and incubated at 37 °C until they reached OD_600_ = 0.500 (4 × 10^8^ CFU/mL). These cells were diluted 100-fold in MHII media. The diluted cells were added to each well in a 96-well plate, affording a final concentration of about 4 × 10^5^ CFU/mL after addition of media and drug.

Compounds were dissolved in either DMSO or dH_2_O, then serially diluted into 1.5 mL microcentrifuge tubes to generate stock solutions. These solutions were prepared such that the final concentration in the wells was no more than 0.5% DMSO. In a 96-well plate, first the media was added followed by the compound of interest. Each well had a final volume of 200 µL. The contents of each well were mixed by pipetting up and down three times. Empty wells were filled with buffer to minimize evaporation, and the edges of the plates were taped to further this end. They were incubated at 37 °C overnight, and the results were collected by measuring the O.D._600_ on a BioTek Cytation 5 plate reader. Percent inhibition was calculated by comparing the well to growth of cells alone. MIC was identified as the concentration which resulted in 90% growth inhibition.

### 3.3. Checkerboard Experiments

In the checkerboard experiment, each column contained a different concentration of amphiphile and each row had a different concentration of antibiotic. In both cases, the concentrations varied by a factor of two. Four controls were employed on each checkerboard plate: full growth (only cells), no growth (only media), and then a column for each drug alone to accurately reflect their individual MIC values. The concentrations of amphiphile and antibiotic tested were ½, ¼, ^1^/_8_, ^1^/_16_, and ^1^/_32_ of the MIC. Preparation of the stock solutions and plates was by the same method described in the minimum inhibitory concentration section.

### 3.4. Membrane Permeability Studies

Membrane permeability studies were conducted as detailed in [48].

### 3.5. pH Study

The pH study was conducted as described in [48].

### 3.6. Growth Curves

The determination of growth curves was performed against *S. aureus* 1199B, which is norfloxacin resistant at 32 µM. Norfloxacin was used at 64 µM as a control. The compounds tested were **1**–**6**, C_10_LE and C_12_LE and their hydrochlorides and methiodides. *S. aureus* 1199B was cultured overnight from a single CFU and reduced to OD_600_ = 0.55 before use. Samples were prepared in LB media to which was added either the norfloxacin control or the combination of norfloxacin and one of the compounds C_10_LE (**1**), C_10_LE•2HCl (**2**), C_10_LE•2MeI (**3**), C_12_LE (**4**), C_12_LE•2HCl (**5**), or C_12_LE•2MeI (**6**). Dimethylsulfoxide (DMSO) was used to solubilize the lariat ether derivative, when necessary, but its concentration was never allowed to exceed 0.5% (*v*/*v*) in all experiments. The samples were vortexed (200 RPM), 20 mL of diluted *S. aureus* 1199B was added, and the sample was vortexed again followed by incubation at 37 °C. The absorbance of each sample (600 nm) was recorded every quarter hour for 24 h. Each growth curve was repeated in triplicate and the average of each data point was used to plot the graph shown in Figure 6.

### 3.7. Antimicrobial Complexation—Visual and NMR

These studies were conducted as previously reported [60].

### 3.8. Dynamic Light Scattering

Measurements were performed on a Brookhaven Instruments Corp. ZetaPALS instrument at 25 °C using a 660 nm laser and correlating scattering at 90°. Samples were prepared by initially dissolving the amphiphile in filtered DMSO obtained from ThermoFisher. Next, they were diluted into 1× PBS such that DMSO was fixed at 0.5% in 15 mL sterile conical tubes. 10× PBS was obtained commercially, then diluted 10-fold in MQ H_2_O. The mixture was vortexed and incubated at room temperature for 2 h, then transferred to a clean quartz cuvette and equilibrated in the instrument for 10 min at 25 °C. Ten measurements consisting of 2 min runs were made on each sample. The average effective diameter was calculated with standard error reported for at least three trials.

### 3.9. Membrane Depolarization

The membrane depolarization study was conducted as described in [48].

### 3.10. Planar Bilayer Voltage Clamp Study

The planar bilayer experiments were conducted as described previously [67].

### 3.11. Efflux Pump Study

Experimental details have been reported in [44].

### 3.12. Cytotoxicity

The cytotoxicity experiments were conducted as described in [44].

## 4. Conclusions

Within the dialkyldiaza-18-crown-6 lariat ether family examined, the compounds having *bis*(decyl) and *bis*(dodecyl) sidearms proved to possess the greatest antimicrobial activity among the free bases. These simple-to-prepare compounds were converted into the corresponding hydrochloride and methiodide salts, with a consequent alteration in their biological activity. In addition to activity against both Gram-negative and Gram-positive bacteria, certain lariat ether derivatives functioned as adjuvants to enhance potency and reverse the resistance of antimicrobials. This was demonstrated by MIC determination and checkerboard studies. The data indicate that the lariat ethers interacted with membranes to enhance permeability (propidium iodide tests, planar bilayer experiments). The lariat ethers inhibited efflux pump function, diminishing the resistance response of bacteria to antimicrobials. They did not affect bacterial growth at sub-MIC levels (growth curves). Combinations of lariat ether and antimicrobial enhanced the potency of the latter (checkerboard experiments). In vitro cytotoxicity studies demonstrated their safety, at least in vitro. The lariat ether free bases complexed with a range of FDA-approved antibiotics, as demonstrated by visual and NMR experiments as well as dynamic light scattering studies. Taken together, the lariat ether derivatives offer significant potential as an antimicrobial adjuvant that can be combined with drugs whose potencies are currently fading.

Based on the experimental results described herein, C_10_LE•2HCl (**2**) and the corresponding MeI salt are good candidates to study further. The C_10_LE•2HCl (**2**) had good solubility and was not affected by changes in the pH. It also penetrated membranes and depolarized them enough to inhibit efflux pump activity, but growth was not inhibited (growth curves). As such, it is a promising adjuvant that can be used in combination with antibiotics that are losing efficacy as a result of efflux pump-related resistance. This was shown with the combination studies and the 1:1 interaction of LEs with antimicrobials. It is of further interest that the cytotoxicity of the MeI salts (separation index of 32-fold) was lower than that of HCl salts (separation index of only 8-fold), making them both strong candidates for further development.

## Figures and Tables

**Table 1:** Minimum inhibitory concentrations (µM) against DH5α *E. coli*.

*LE Sidearms*	*LE*	*LE•HCl*	*LE•MeI*
*n*-octyl	>128	>128	>128
*n*-decyl	11	4	24
*n*-undecyl	20	nd ^a^	nd
*n*-dodecyl	>128	>128	2
*n*-tetradecyl	>128	>128	>128

^a^ Not determined.

**Table 2:** Solubility limits (µM) of compounds **1**–**6**.

Compounds	MHII ^a^	PBS ^b^
Verapamil	>128	>128
Reserpine	16	16
C_10_LE (**1**)	≥128	≥128
C_10_LE•2HCl (**2**)	8	8
C_10_LE•2MeI (**3**)	64	≥128
C_12_LE (**4**)	16	16
C_12_LE•2HCl (**5**)	4	2
C_12_LE•2MeI (**6**)	1–2	≥128

^a^ MH II = Mueller Hinton II. ^b^ PBS = Phosphate-buffered saline.

**Table 3:** MICs (µM) of LEs and antibiotics.

Compounds Used	K12 *E. coli*	Tet^R^ *E. coli*	*S. aureus* 1199B
Norfloxacin	0.125	nd	64
Tetracycline•HCl	2	1000	nd
C_10_LE (**1**)	10	8	8
C_10_LE•2HCl (**2**)	4	8	2
C_10_LE•2MeI (**3**)	16	32	1–2
C_12_LE (**4**)	>128	>64	32
C_12_LE•2HCl (**5**)	>128	>64	4
C_12_LE•2MeI (**6**)	4	16	1

nd: not determined.

**Table 4:** MICs (µM) of LEs against *S. aureus* under various conditions.

Compounds Used	MHII			MH	MH + Ca^2+^
	pH 6.4	pH 7.4	pH 8.4		
C_10_LE (**1**)	16	≥16	32	16	16
C_10_LE•2HCl (**2**)	2	2	4	2	8
C_10_LE•2MeI (**3**)	2	2	2	2	16
C_12_LE (**4**)	>16	>32	>64	nd	nd
C_12_LE•2HCl (**5**)	4	4	>64	nd	nd
C_12_LE•2MeI (**6**)	2	2	2	nd	nd

nd: not determined.

**Table 5:** Complexation ratio between tetracycline derivatives and C_10_LE in CDCl_3_^a^ as determined by ^1^H-NMR.

Antimicrobial	Complexation Ratio ^b^
Tetracycline•HCl	1.03 ± 0.02
Minocycline•HCl	1.06 ± 0.05
Doxycycline•HCl	1.02 ± 0.04
Oxycycline•HCl	0.997 ± 0.01
Chlortetracycline•HCl	No complexation observed

^a^ Identical results within experimental error were obtained using either CDCl_3_ or CD_2_Cl_2_ as the NMR solvent. ^b^ All ratios were determined at least in triplicate.

## Data Availability

Data may be obtained by email to the corresponding author.
